# A team approach to the transfer of robotics from technical services to a QC environment

**DOI:** 10.1155/S1463924696000132

**Published:** 1996

**Authors:** Joseph R. Tricome, Naseem Muhammad, Stanley W. Swieck, James J. Rice

**Affiliations:** Bristol-Myers Squibb Company PO Box 4755 Syracuse NY 13221-4755 USA


Journal of Automatic Chemistry, Vol. 18, No. 4 (July–August 1996), pp. 143–146

### A team approach to the transfer of robotics from technical services to a QC environment

**Joseph R. Tricome, Naseem Muhammad, Stanley W. Swieck, Jr. and James J. Rice**
*Bristol-Myers Squibb Company, PO Box 4755, Syracuse, NY 13221-4755, USA*

*In today's competitive environment in the pharmaceutical industry, and with increased emphasis on productivity and efficiency, a team approach is essential to successful implementation of a robotic system in a quality control (QC) environment. A QC initiative was undertaken at Bristol-Myers Squibb in Syracuse to implement a robotic system to analyse penicillin fermentation broths on a 24-hour basis. This team effort has resulted in the introduction of robotics to the QC laboratories and a smooth transition to a fully automated analysis technique.*

### Introduction

In June 1994, a project was undertaken by Technical Services at Bristol-Myers Squibb (BMS) in Syracuse, NY, to develop a robotic procedure for the HPLC analysis of penicillin broths for the quality control (QC) laboratory. The manual method used was both time consuming and dependent on a full time staff of technicians being available to test samples. Since the QC operation was only two shifts, this resulted in an eight hour time period where no analysis of fermentation broths was being performed. As a result of the lack of analysis on one shift, long turnaround time for analysis of the penicillin fermentation time points and minimum analysis capacity, the feasibility of developing a robotic procedure was explored. Using a team approach of technical personnel from QC technical services, and Zymark, a cost-effective, high-efficiency, robotic procedure was developed and implemented for the analysis of penicillin broths received from fermentation production.

### Method of analysis

The manual method of analysis originally developed for the penicillin fermentation broth, was very labour intensive and required two HPLC instruments to test the 20 to 40 broth samples per day. The data analysis of the fermentation sample took an additional one to two hours to process the results and forward the information to the fermentation department. In addition, no samples of the fermentation tanks were being submitted after 11 p.m., because there were no analytical services available. The manual method did not provide the efficiency or turnaround needed to provide service to the fermentation department. In order to resolve the problem and take full advantage of an unattended robotic analysis system, the following criteria were used as guidelines for development of an automated system. The robotic system had to handle the following:

(1) Short HPLC analysis time/short preparation time.
(2) On-line computer processing of data.
(3) Sample analysis on a 24-hour basis.
(4) Trained laboratory staff.
(5) Minimal down time.

### HPLC analysis time

The manual method was taking about 20 minutes to analyse a single sample. This run time, if implemented, for the robotic method, would not provide any advantage, with regard to faster response to the fermentation department. The HPLC development chemists of the technical services section were involved with the project and worked closely with the robot development team to make recommendations for an alternative HPLC method. The new method developed for the robotic analysis had a total run time of only 10 minutes and was based on a high efficiency $C_{18}$ μBondapak 15 cm column (Waters Corporation, Milford, MA, USA), which resolved impurities and quantified the penicillin content in the fermentation broth. This HPLC method was shown to be rugged, reproducible, specific, accurate and precise.

### Results processing

The QC laboratory using the manual method of analysis would routinely analyse a set of broth samples during each shift and report the data on the next shift. This resulted in a lag time of about 8 to 14 hours between data points on a fermentation tank. The robotic procedure reduces the run time by about half. Once the data is generated on-line, and reviewed, the result can be electronically available to the fermentation department. Because of the fast HPLC robot procedure, data can be provided in four hours for monitoring of the fermentation tanks, as compared to the previous eight to 14 hours.

### Sample analysis

Sample analysis of the penicillin fermentation broth was performed by the QC analyst manually preparing the sample volumetrically, performing a serial dilution, and a final filtration step. This work was performed on the first and second shifts each day of the year. Samples taken on the third shift were refrigerated and analysed on first shift.

0142-0453/96 $12.00 © 1996 Taylor & Francis Ltd.

The robotic method developed, on a Zymark XP Robotic System using PyTechnology (Zymark Corporation, Hopkinton, MA) was designed for 24-hour operation. The fermentation operator takes a broth sample at the scheduled time in a plastic tube and places a bar code on the tube. The bar code information includes the tank number, age in hours of the sample, and the lot number for the batch. These samples are then taken to the robot, which is located in a separate room near the fermentation area, and places the tube in a chilled rack located on a Pysection rack of the robot. The robot picks up the sample and performs the sample preparation and analysis. If there is no sample tube for the robot to pick up, the system will wait a period of time then try to pick up a tube at the same position again, until it actually has a sample tube to prepare.

This robotic system would provide immediate feedback to the fermentation department after sample analysis on a 24 hour basis. Figure 1 shows the layout of the Zymark robotic system designed for the analysis of penicillin broths.

### Trained laboratory staff

The development of the Zymark robotic system for analysis of penicillin broths was a team approach. Initially, the technical services group designed and programmed a robotic set-up and started the initial developmental stage of the system using the fast HPLC method. Once the feasibility studies were completed, a meeting was held with QC to discuss the project and provide an overview of the robotic system. The robotic system, set up in technical services for the feasibility studies, was also demonstrated to show the consistency of an actual working system. An order was then placed to Zymark for a new robotic system to be assembled according to the technical services department's design specifications. The program written for the robotic system, to assay the broths, was then provided to Zymark and a representative from technical services went to Zymark to complete the operational set-up of the system. To assure success of the project, a QC supervisor and a senior group leader, were sent to Zymark for the Basic Pytechnology course. Completion of the robotic system's assembly, at Zymark, was co-ordinated to coincide with the attendance of the QC personnel. After completion of the basic course, the QC people stayed an extra two days to familiarize themselves with the operation of the robot. The system was then installed in the QC area at BMS. Working closely with Zymark, another training programme, which would be given at BMS, was designed to introduce and instruct other QC personnel on the operation of the robotic system. The training programme, designed by the Zymark Center

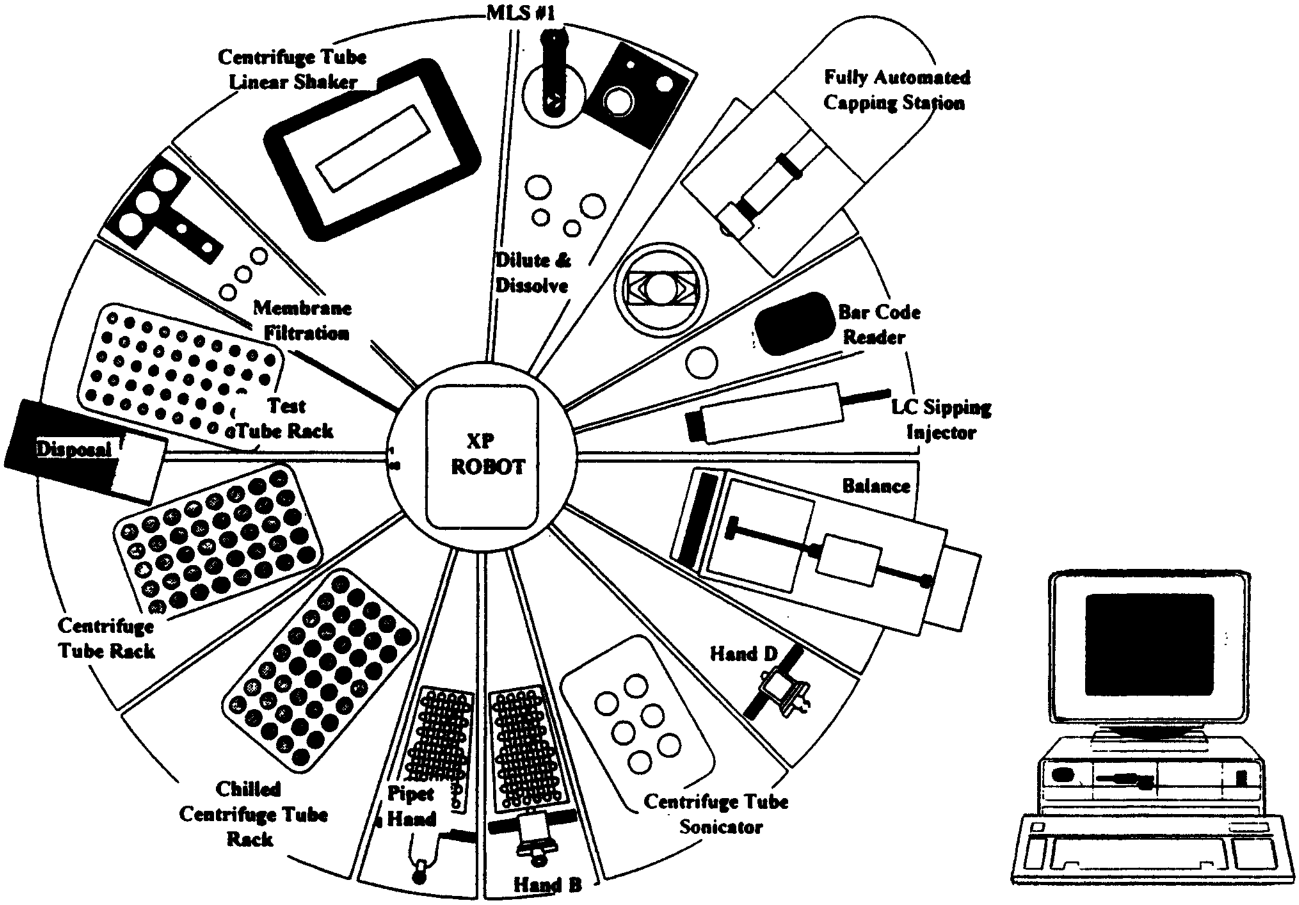

*Figure 1. Zymark robotic system for the analysis of penicillin broths.*

for Training and Development in conjunction with the robot design team at BMS, instructed the QC personnel on the fundamental operation of the system. Also, this training was to provide the necessary skills for the QC technician to solve minor operational difficulties associated with the system on a daily basis. This week-long course provided a fundamental introduction to the Zymate Laboratory Automation System and was not designed for in-depth development of robotic assays. Each QC technician had the opportunity to work with the system and familiarize themselves with the broth application. The training also included troubleshooting contrived situations, teaching positions, handling the data and minor maintenance.

### System validation

The validation of the robotic system for analysis of penicillin broths involved several stages. The validation protocol entailed: system operational validation; program validation; liquid transfer validation; and bridge studies.

### *System operational validation*

System operational validation involved the validation of the robotic system's individual module's functions, as well as the total functionality of the system as a unit. As part of the validation/qualification process, error checking by the system was also incorporated into the validation. A typical example, shown in figure 2, is a section of validation for a rack.

### *Program validation*

Program validation involved the validation of the programming steps used for the analysis of the broth. This validation is critical to the overall operation of the robot because the execution of the movements of the robot incorporates both the system programs, as well as newly derived programs written specifically for the application. An example of the program qualification write up is given in figure 3, for a segment of derived programming.

### *Liquid transfer validation*

The first stage of the liquid transfer validation is based on the qualification of the system to transfer aliquots and deliver diluent volumes. These verifications are based on the gravimetric delivery of a programmed amount of liquid, typically water. The system is first programmed to transfer different aliquots of a liquid using the pipette hand installed on the system. These transfer aliquots are 25%, 50%, 75% and 100% of the maximum deliverable volume of the pipette hand. Each aliquot is repeated five times and analysed to ensure that the resultant delivery is within GMP/USP guidelines. Each syringe on the master laboratory station (MLS), which would be used in a quantifiable situation in an application, is then programmed to deliver 50% and 100% of its maximum allowed volume. Again, as with the transfer aliquots, each delivery is repeated five times and analysed to ensure that the resultant delivery is within GMP/USP guidelines. This liquid transfer validation insures proper functioning of all pertinent transfer capabilities of the system.

The second stage of the liquid transfer validation is based on the qualification of the system's ability to aliquot the proper amount of sample and deliver the proper amount of the prescribed diluent within the operation of the broth application. A typical example of the MLS delivery of the broth diluent is shown in table 1.

This represents a 99·8% recovery using 45·0 ml as the programmed deliverable volume. The sample aliquot ability of the system is also qualified. A typical example of the system aliquot transfer within the program is shown in table 2.

This represents a 100·4% recovery using 3·00 ml as the programmed aliquot volume. With each instance of any liquid aliquot or dilution value specified in the application the operation of the system is verified accordingly. These values are continuously monitored during the processing of a sample and are printed out and/or saved to disk

*Table 1. Standard diluent (ml).*

| | |
|---|---|
| | 44·9326 |
| | 44·9222 |
| | 44·9133 |
| | 44·9010 |
| | 44·9210 |
| $Mean_5$ = | 44·9180 |
| Standard deviation = | 0·0117 |
| Relative standard deviation = | 0·03% |

```

MODULE NAME:            RACK 1 (50cc test tube. 40 positions) - R1:

Module Serial Number:   N.A.

    MANUAL CONTROL (Z > Prompt), Rack 1 - use HAND.B

    NOTE:  Any Failed Movement Requires System Trouble Shooting - Stop Verification - Module Fails

    Comment                Expected Results                                 Observed Results (YES or FAIL)

    Z > R1: INIT           Screen shows, "Initializing RACK.1...please wait"     ______

                           Z > prompt.

    Z > RACK.1.INDEX=38    No action taken by system returns Z > prompt         ______

    Z > MOVE.OVER.RACK.1   System halt, ERROR "There is NO hand..."             ______

                           RECOVERY

                           Follow instructions on screen, robot moves to

                            position over RACK.1, position 38.  Z > prompt      ______

```

*Figure 2. Validation of a rack.*

| | | |
|---|---|---|
| 2. | SET.PARAMETERS.BROTH | |
| | A. - Statement "Are you restarting ..." prints to screen - | Question answer Y - Program goes to #1, 2G1<br>Question answer N - continue<br>Any other Alpha answer except Y or N will cause an "Invalid Response" error any other Numerical entry (besides 0 or 1) will cause the screen to reprint<br>a. Progam Function Correctly |
| | B. - INITIALIZE SYSTEM | System messages print on screen stating that modules are being initialized<br>a. Program Function Correctly |
| | C. - PURGE | All MLS syringes purged with solvent - syringes draw full length and dispense to origin.<br>a. Program Function Correctly |
| | D. - WASH.LC.INJECTOR | HPLC sipper injector is washed - Syringe A is drawn and dispensed to origin through sipper tube. |

*Figure 3. Program qualification.*

*Table 2. Sample aliquot (ml).*

| | |
|---|---|
| | 3·0088 |
| | 3·0139 |
| | 3·0107 |
| | 3·0160 |
| | 3·0134 |
| $Mean_5$ = | 3·0126 |
| Standard deviation = | 0·0028 |
| Relative standard deviation = | 0·09% |

*Table 3. Typical bridge between manual and robotic data.*

| Sample No. | Manual method (μgm/mg) | Robotic method (μgm/mg) |
|---|---|---|
| 1 | 1 416 | 1 390 |
| 2 | 1 712 | 1 738 |
| 3 | 22 653 | 23 582 |
| 4 | 29 304 | 29 344 |
| 5 | 32 799 | 32 927 |

file, with the other pertinent sample information upon injection of the HPLC broth sample for analysis.

*Data storage and transfer*

All pertinent information inputted and/or generated by the system is either printed out upon HPLC injection and/or is transferred to file within the computer system. Data transfer by the robotic system was initially qualified during the system operational validation. To ensure correct generation of data and proper management of data by the system, during the application, critical values and sample information are printed out during the system's operation. The generation of the data was also initially verified during the program validation portion of the system's qualification protocol. All data is then checked at the termination of a broth application, by the QC technician, to verify correct operation of the system.

*Bridge studies*

The ultimate proof of system's performance and evaluation is a bridge study comparing the results of the same samples assayed by the robotic HPLC procedure and the manual HPLC procedure. If the data indicates that there are no statistically significant differences between the results of the two methods, and the proper aliquot and dilution values were used in preparing the sample, the entire validation procedure would be acceptable. A typical bridge between the manual and the robotic data is shown in table 3.

### Conclusion

A Zymark automated method was successfully developed, validated and implemented in the quality control department at Bristol-Myers Squibb, for the analysis of penicillin fermentation broths. The integration of the technical method development, coupled with the co-operative effort of QC and Zymark has resulted in an efficient, high volume method for increased productivity.

This project would not have been accomplished without a team approach. The diverse backgrounds of people involved as well as Zymark's training resources were critical to the final implementation of the system.

